# Supplementary material for: PomX, a ParA/MinD ATPase activating protein, is a triple regulator of cell division in Myxococcus xanthus
Source: eLife. 2021 Mar 18;10:e66160. doi: 10.7554/eLife.66160 (PMC7993993; doi:10.7554/eLife.66160)
Supplement: Transparent reporting form [file elife-66160-transrepform.docx]

***eLife’s* transparent reporting form**

We encourage authors to provide detailed information *within their submission* to facilitate the interpretation and replication of experiments. Authors can upload supporting documentation to indicate the use of appropriate reporting guidelines for health-related research (see [EQUATOR Network](http://www.equator-network.org/%20)), life science research (see the [BioSharing Information Resource](https://biosharing.org/)), or the [ARRIVE guidelines](http://www.plosbiology.org/article/info:doi/10.1371/journal.pbio.1000412) for reporting work involving animal research. Where applicable, authors should refer to any relevant reporting standards documents in this form.

If you have any questions, please consult our Journal Policies and/or contact us: [editorial@elifesciences.org](mailto:editorial@elifesciences.org).

**Sample-size estimation**

- You should state whether an appropriate sample size was computed when the study was being designed
- You should state the statistical method of sample size computation and any required assumptions
- If no explicit power analysis was used, you should describe how you decided what sample (replicate) size (number) to use

No statistical methods were used to predetermine the sample size. The sample size was determined based on our expertise in bacterial cell biology. Generally, at least two biological replicates with a high number of cells (n) were used for the image analysis of fluorescence pictures. Similarly, we decided that based on our own experience, for biochemical experiments, several independent protein purifications served as biological replicates.

Please outline where this information can be found within the submission (e.g., sections or figure legends), or explain why this information doesn’t apply to your submission:

**Replicates**

- You should report how often each experiment was performed
- You should include a definition of biological versus technical replication
- The data obtained should be provided and sufficient information should be provided to indicate the number of independent biological and/or technical replicates
- If you encountered any outliers, you should describe how these were handled
- Criteria for exclusion/inclusion of data should be clearly stated
- High-throughput sequence data should be uploaded before submission, with a private link for reviewers provided (these are available from both GEO and ArrayExpress)

Please outline where this information can be found within the submission (e.g., sections or figure legends), or explain why this information doesn’t apply to your submission:

Data shown for fluorescence microscopy, live-cell imaging, transmission electron microscopy, immunoblot experiments, sedimentation assays and in vitro pull-down experiments were obtained in at least two independent experiments with similar results.

Localization patterns from fluorescence microscopy data collection are representative and the n-value is given in the figures or figure legends.

For qualitative determination of protein-protein interactions using the BACTH assays for any given combination, three independent clones were analyzed. Protein purification and SDS-PAGE analysis were performed several times with similar results. ATPase activity was determined from multiple independent experimental measurements as mentioned in the figure legend.

No data were excluded from the data analysis.

**Statistical reporting**

- Statistical analysis methods should be described and justified
- Raw data should be presented in figures whenever informative to do so (typically when N per group is less than 10)
- For each experiment, you should identify the statistical tests used, exact values of N, definitions of center, methods of multiple test correction, and dispersion and precision measures (e.g., mean, median, SD, SEM, confidence intervals; and, for the major substantive results, a measure of effect size (e.g., Pearson's r, Cohen's d)
- Report exact p-values wherever possible alongside the summary statistics and 95% confidence intervals. These should be reported for all key questions and not only when the p-value is less than 0.05.

Please outline where this information can be found within the submission (e.g., sections or figure legends), or explain why this information doesn’t apply to your submission:

Statistical analysis methods used in this study are described in the methods section. If appropriate the mean, the median and the standard deviation are shown in the figure or the figure legend. Box plots, for visualization of data, are described in the figure legends. If appropriate, p-values are indicated in the figures and are explained in the figure legends.

(For large datasets, or papers with a very large number of statistical tests, you may upload a single table file with tests, Ns, etc., with reference to sections in the manuscript.)

**Group allocation**

- Indicate how samples were allocated into experimental groups (in the case of clinical studies, please specify allocation to treatment method); if randomization was used, please also state if restricted randomization was applied
- Indicate if masking was used during group allocation, data collection and/or data analysis

Please outline where this information can be found within the submission (e.g., sections or figure legends), or explain why this information doesn’t apply to your submission:

Allocation into experimental groups does not apply to our study. Masking or blinding was not relevant for our study.

**Additional data files (“source data”)**

- We encourage you to upload relevant additional data files, such as numerical data that are represented as a graph in a figure, or as a summary table
- Where provided, these should be in the most useful format, and they can be uploaded as “Source data” files linked to a main figure or table
- Include model definition files including the full list of parameters used
- Include code used for data analysis (e.g., R, MatLab)
- Avoid stating that data files are “available upon request”

Please indicate the figures or tables for which source data files have been provided:

The source data underlying Figure 1A, B, D, 2A, B, C, D, E, F, G, H, I, 3A, B, D, 4A, B, C, 5A, B, C, D, E, 6B, 7 and Figure 1_Figure supplement 1B, Figure 2_Figure supplement 1A-F, Figure 4_Figure supplement 1A, B and Figure 4_Figure supplement 2A, B are provided as a Source Data file and include uncropped and unprocessed scans of all western blots, SDS-PAGE gels, TEM picture and BACTH plate scans as well as data from ATPase assays.
